# Porcine Complement Regulatory Protein CD46 Is a Major Receptor for Atypical Porcine Pestivirus but Not for Classical Swine Fever Virus

**DOI:** 10.1128/JVI.02186-20

**Published:** 2021-04-12

**Authors:** Gökce Nur Cagatay, Aleksandra Antos, Oliver Suckstorff, Olaf Isken, Norbert Tautz, Paul Becher, Alexander Postel

**Affiliations:** a Institute for Virology, University of Veterinary Medicine Hannover, Hannover, Germany; b Department of Virology, National Veterinary Research Institute, Puławy, Poland; c Klifovet AG, Munich, Germany; d Institute of Virology and Cell Biology, University of Luebeck, Luebeck, Germany; University of Southern California

**Keywords:** atypical porcine pestivirus, classical swine fever virus, complement regulatory protein CD46, host tropism, pestiviruses, receptor, virus entry

## Abstract

Pestiviruses such as bovine viral diarrhea virus (BVDV) and classical swine fever virus (CSFV) belong to the family *Flaviviridae* and represent pathogens of outstanding veterinary relevance. Pestiviruses enter cells via receptor-mediated endocytosis. For entry in bovine cells, complement regulatory protein CD46_bov_ serves as a cellular receptor for BVDV. In this study, the role of porcine CD46_pig_ in cellular entry was investigated for the recently discovered atypical porcine pestivirus (APPV), CSFV, and Bungowannah virus (BuPV) in order to elucidate the observed differences in host cell tropism. A cell culture-adapted APPV variant, which shows enhanced viral replication *in vitro*, was generated and demonstrated a strict tropism of APPV for porcine cells. One of the porcine cell lines displayed areas of CD46_pig_-expressing cells and areas of nonexpressing cells, and one single cell line revealed not to express any CD46_pig_. The CD46_pig_-deficient porcine lymphoma cell line, known to facilitate CSFV replication, was the only porcine cell line nonpermissive to APPV, indicating a significant difference in the entry mechanism of APPV and CSFV. Infection experiments with a set of genetically engineered CD46_pig_ knockout cells confirmed that CD46_pig_ is a major receptor of APPV as CD46_bov_ is for BVDV. In contrast, it is apparently not an essential determinant in host cell entry of other porcine pestiviruses such as CSFV and BuPV. Existence of a CD46_pig_-independent entry mechanism illustrates that the pestiviral entry process is more diverse than previously recognized.

**IMPORTANCE** Pestiviruses comprise animal pathogens such as classical swine fever virus (CSFV) and bovine viral diarrhea virus (BVDV) that cause notifiable diseases with great economic impact. Several additional pestivirus species affecting animal health were recently identified, including atypical porcine pestivirus (APPV). APPV is associated with health problems in piglets and is highly abundant in pig populations worldwide. Complement control protein CD46 serves as a receptor for diverse bacterial and viral pathogens, including particular adenoviruses, herpesviruses, measles virus (MeV), and BVDV. Porcine CD46 (CD46_pig_) was suggested to be a major receptor for CSFV. Here, we identified remarkable differences in relevance of CD46_pig_ during entry of porcine pestiviruses. Resembling BVDV, efficient APPV infection in cell culture depends on CD46_pig_, while other porcine pestiviruses can efficiently enter and infect cells in the absence of CD46_pig_. Thus, the study provides insights into the entry process of these pathogens and may help to understand differences in their biology.

## Abstract (precis)

Pestiviruses comprise animal pathogens such as classical swine fever virus (CSFV) and bovine viral diarrhea virus (BVDV) that cause notifiable diseases with great economic impact. Several additional pestivirus species affecting animal health were recently identified, including atypical porcine pestivirus (APPV).

## INTRODUCTION

The genus *Pestivirus* belongs to the family *Flaviviridae* and currently comprises 11 different species termed *Pestivirus A* to *K* and a growing number of putative new species (1). Pestiviruses such as bovine viral diarrhea virus (BVDV) and classical swine fever virus (CSFV) are RNA viruses of outstanding veterinary and economic relevance, being causative agents of notifiable diseases (2, 3). Porcine host species are significantly implicated in pestivirus biology. CSFV (*Pestivirus C*) is the only established pestivirus selectively infecting porcine hosts. Moreover, pigs are susceptible for several pestivirus species, including ruminant pestiviruses like BVDV type 1 and 2 (*Pestivirus A* and *B*) and Border disease virus (BDV, *Pestivirus D*). In addition to CSFV, three additional porcine pestivirus species were discovered, comprising atypical porcine pestivirus (APPV, *Pestivirus K*), Bungowannah virus (BuPV, *Pestivirus F*), and LINDA virus (LindaV, species not yet approved). These three pestivirus species can cause severe diseases in young piglets (4–6). It appears likely that infections of pigs with LindaV and BuPV were results of rare or even unique spillover infections from so far unknown reservoir hosts since subsequent attempts to detect these pathogens in domestic pig and wild boar populations failed (5–8). In contrast to BuPV and LindaV, APPV was found to be highly abundant in domestic pigs from many countries worldwide, and wild boar must be considered a natural reservoir host (9, 10). Pestivirus genomes that are most closely related to APPV were detected in bats, which together with rodents represent the first non-ungulate hosts harboring novel pestivirus species (11–13). So far, it is not known whether pigs are the only host for APPV, and investigation of *in vitro* cell tropism was restricted due to lack of an APPV cell culture isolate. In contrast to that of BuPV and LindaV, APPV isolation and propagation is highly inefficient on established porcine cell lines, and virus isolation was only reported on embryonic porcine kidney epithelial cells (SPEV cells) with very low infectious titers (14, 15).

Although it is a crucial step in the viral replication cycle, the entry process of pestiviruses is still poorly understood. Pestiviruses enter the host cell by receptor-mediated endocytosis, likely via a multistep process using different host cell factors (16, 17). Historically, bovine complement control protein CD46 (CD46_bov_) was identified as a receptor for BVDV by a set of neutralizing monoclonal antibodies (mabs) that were directed not against the viral antigen but against the cellular protein (18, 19). CD46 was previously named a “pathogen’s magnet,” as it is used as a receptor by diverse bacterial and viral pathogens, including measles virus (MeV) vaccine strains, certain human adenoviruses, and herpesviruses such as human cytomegalovirus (20, 21). Expression of CD46_bov_ on porcine cells increases the susceptibility to BVDV (18). Nevertheless, BVDV was not able to infect nonpermissive cells substituted with CD46_bov_. As CD46_bov_ is an almost ubiquitously expressed molecule, it remained unclear what determines tropism of BVDV for certain tissues. One possible explanation would be the use of coreceptors or different CD46_bov_ variants by the virus (20, 22). Evidence for an additional BVDV receptor was provided by a study which demonstrated cellular entry independent from binding of E2 to CD46_bov_ (23). Subsequently, porcine CD46 (CD46_pig_) was suggested to play a role in the entry process of CSFV, as CD46_pig_-specific monoclonal antibodies (mabs) diminished infectivity to some degree (24).

In the present study, we systematically investigated the role of CD46_pig_ for entry of the porcine pestivirus species APPV, CSFV, and BuPV. The results of our study show that CD46_pig_ is essential for efficient entry of APPV, while CSFV and BuPV rely on different host cell factors for cellular entry.

## RESULTS

### Adaptation of atypical porcine pestivirus (APPV) to cell culture conditions.

**FIG 1:**
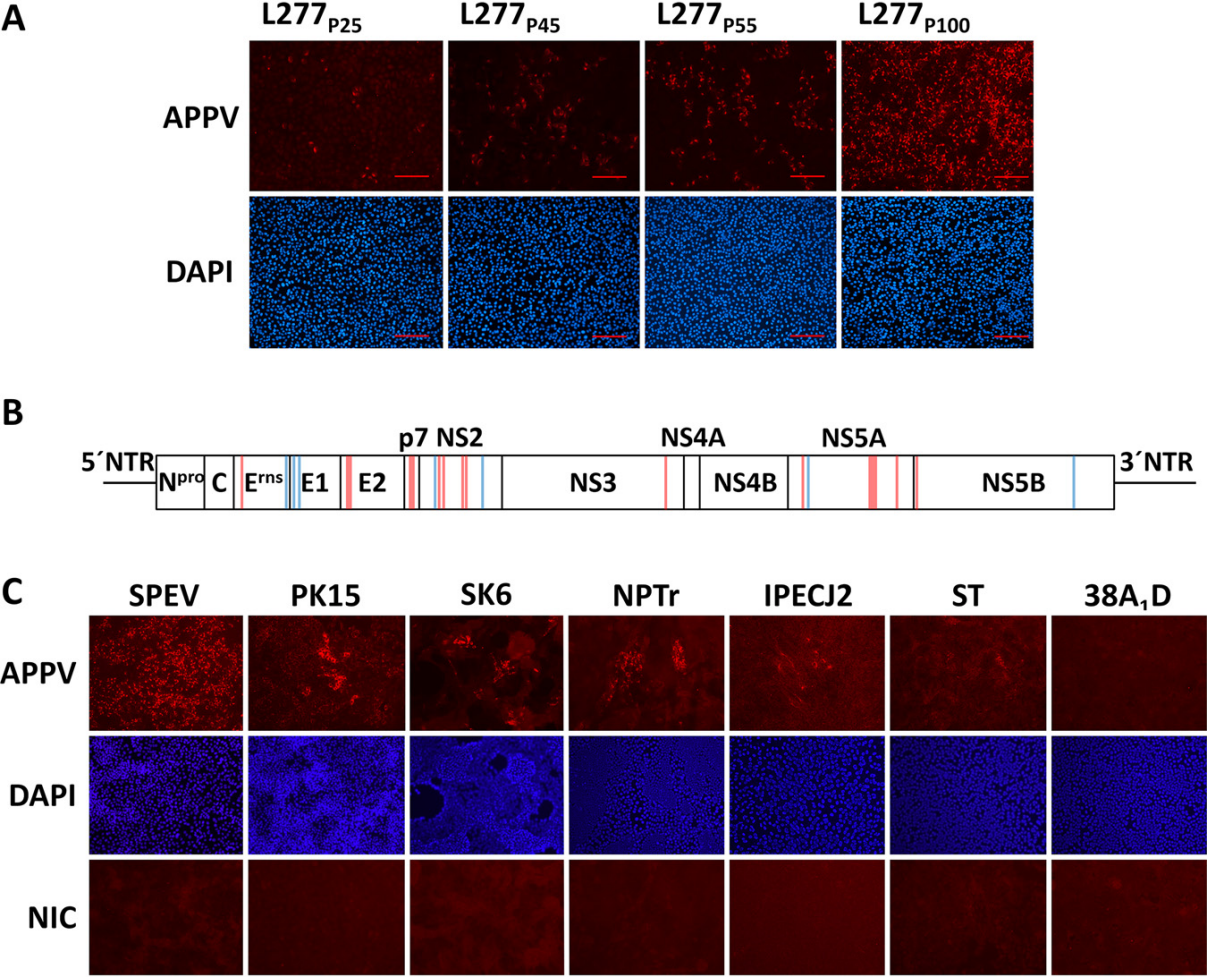
**Adaptation of APPV to cell culture conditions and permissivity of different porcine cell lines to APPV.** (A) SPEV cells were infected with supernatant from the indicated passages (P25, P45, P55, and P100) of SPEV cells persistently infected with APPV isolate L277. Scale bars indicate 200 µm. (B) Schematic representation of the 100th passage of APPV isolate L277 (APPV_P100_) genome including locations of synonymous (blue) and nonsynonymous mutations (red). (C) The cell culture-adapted APPV_P100_ was used to infect different porcine cell lines with an MOI of 1. All porcine cell lines except 38A_1_D were permissive to APPV, showing either nearly complete APPV-infected monolayer (SPEV) or only infected foci (PK15, SK6, NPTr, IPECJ2, and ST). The respective noninfected cells (NIC) served as a control. Immunofluorescence staining was performed at 72 h p.i. using porcine APPV-specific antiserum (red) and DAPI (blue).

Determination of the complete polyprotein coding sequence of APPV from the 100th passage (APPV_P100_) using next-generation sequencing revealed a total of 23 nucleotide exchanges compared to the original sequence obtained directly from the sample material (GenBank MF167291). These genome alterations consist of 7 synonymous and 16 nonsynonymous mutations (Fig. 1B). Three synonymous and three nonsynonymous substitutions, namely, H330Q (E^rns^), N751K (E2), and D752N (E2), were located within the predicted glycoprotein coding regions (Fig. 1B). Sanger sequencing of viral genomes obtained from different APPV passages confirmed that these mutations were acquired during passaging, between the 45th and 100th passages. APPV stock from the 17th passage (APPV_P17_) did not show any mutations within the predicted glycoprotein coding regions. In five independent repetitions of passaging, the same synonymous and nonsynonymous mutations occurred within the predicted glycoprotein coding regions and correlated with an increased number of infected SPEV cells, suggesting their importance for the adaptation of APPV to cell culture conditions.

### Permissivity of different cell lines to atypical porcine pestivirus (APPV).

We investigated the permissivity of different porcine and nonporcine cell lines to APPV infection using the newly established cell culture-adapted APPV strain (APPV_P100_). All porcine cell lines except 38A_1_D (lymphoma) were susceptible to APPV_P100_ infections, however, to various extent (Fig. 1C). This is in line with the situation *in vivo*, as APPV can be detected in cells of diverse tissues and different cell types of infected piglets (25). APPV_P100_ replicated most efficiently in SPEV (kidney) cells, resulting in an almost completely infected monolayer of SPEV cells at 72 h postinfection (p.i.) after infection with an MOI of 1. In contrast, on PK15 (kidney) cells, which are routinely used for propagation of CSFV, only few APPV_P100_-infected foci were observed under equal conditions. Similar to that on PK15 cells, inefficient and limited viral replication of APPV_P100_ was observed on porcine SK6 (kidney), NPTr (neonatal trachea), IPECJ2 (jejunum), and ST (testis) cells. These results demonstrate so far unknown differences in the permissivity of different porcine cell lines to APPV infection (Fig. 1C).

Several nonporcine cells (MDBK, CRFK, RK13, Vero76, Lovo, HypNi/1.1, EidNi/41; Table 1) were also tested for their permissivity to APPV, since BuPV was shown to have a broad cell tropism *in vitro* and sequences of pestiviruses closely related to APPV were detected in diverse rodent and bat species (11–13). None of the investigated nonporcine cell lines was permissive to APPV_P100_ (data not shown). Thus, the data provide no evidence that APPV can efficiently infect and replicate in nonporcine cell lines.

**TABLE 1:** Cell lines used in this study

Species	Name	Tissue	Source/reference
Pig	SPEV	Embryonic kidney	Cell line 0008, FLI, Germany
Pig	PK15	Kidney	Cell line 5-1, FLI, Germany
Pig	SK6	Kidney	Institute’s collection
Pig	38A_1_D	Lymphoma	(45)
Pig	NPTr	Neonatal trachea	(29)
Pig	ST	Testis	Institute’s collection
Pig	IPECJ2	Jejunum	ACC-701, DSMZ
Cattle	MDBK	Kidney	ATCC: CCL-22
Cat	CRFK	Kidney	Institute’s collection
Rabbit	RK13	Kidney	Institute’s collection
African green monkey	Vero76	Kidney	Institute’s collection
Hammer-headed fruit bat	HypNi/1.1	Kidney	(25)
Straw-colored fruit bat	EidNi/41	Kidney	(46)
Human	Lovo	Colon adenocarcinoma	ATCC: CCL-229
Human	HEK293T	Embryonic kidney	ACC-635, DSMZ

### Characterization of CD46_pig_ expression patterns in porcine cell lines.

**FIG 2:**
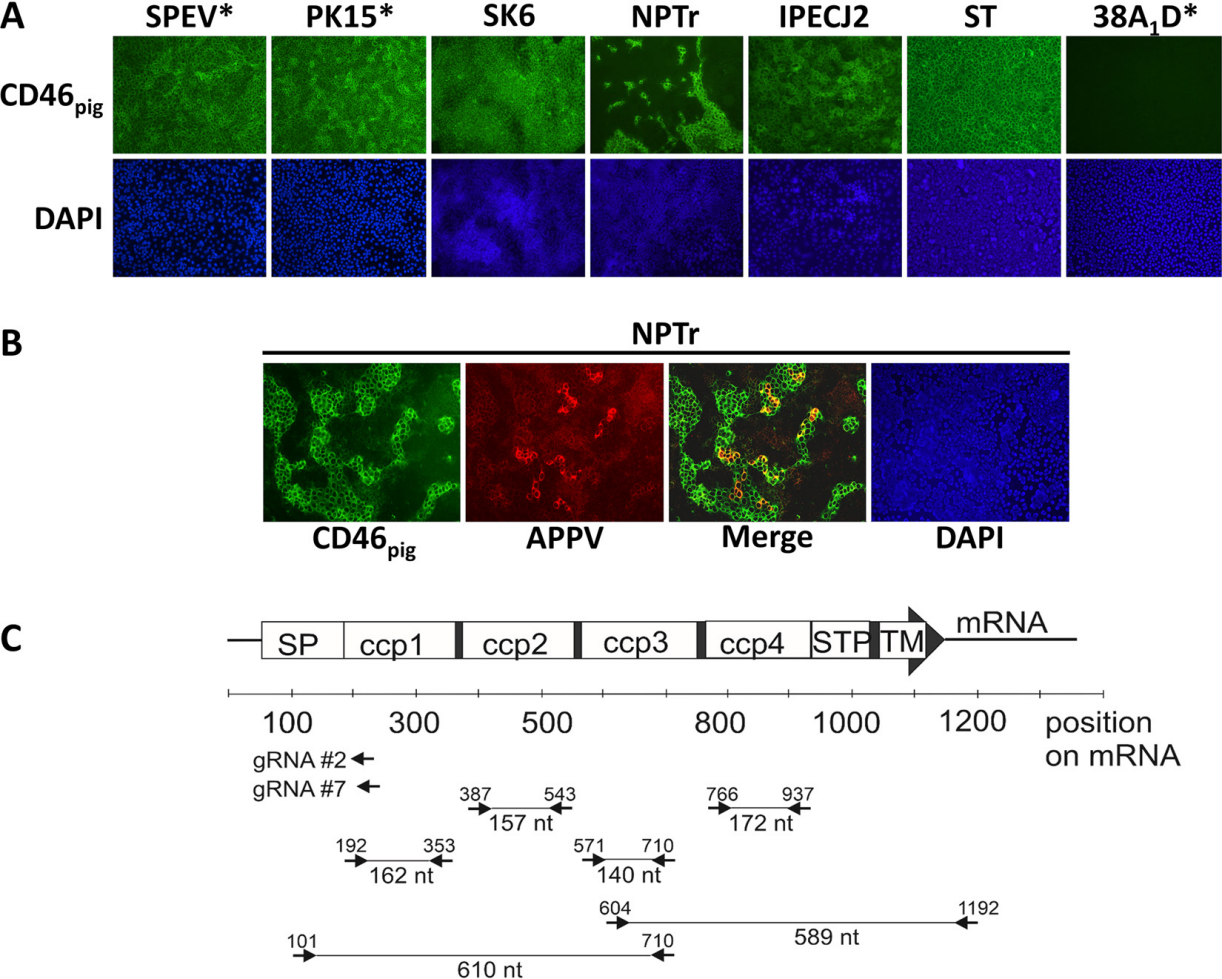
**Characterization of porcine cell lines with regard to their CD46_pig_ expression.** (A) Phenotypical characterization of porcine cell lines by immunofluorescence staining using a CD46_pig_-specific mab (green, MCA2310GA) and DAPI (blue). Asterisks (*) indicate cell lines subjected to conventional RT-PCR for subsequent sequencing. (B) Immunofluorescence staining of APPV_P100_ (porcine APPV-specific antiserum, red), CD46_pig_ (green, MCA2310GA), and DAPI (blue) at 72 h after infection of NPTr cells. (C) Strategy used for genetic characterization and manipulation of the CD46_pig_ gene locus. The CD46_pig_-encoding mRNA was amplified by two RT-PCRs (101/710 and 604/1192) for subsequent cloning and sequencing. Absence of a CD46_pig_-encoding mRNA in porcine lymphoma cell line 38A_1_D was confirmed by RT-PCRs targeting the individual CD46_pig_ domains (primer pairs: 192/353, 387/543, 571/710, 766/937). Positions of signal peptide (SP), complement control proteins 1 to 4 (ccp1-4), serine, threonine, proline-rich region (STP), and transmembrane domain (TM) encoded by the mRNA are depicted. In addition, positions of guide RNAs (gRNA CD46-2 and -7) used for construction of CD46_pig_ knockout cells are indicated (for details see Fig. 3B).

The observed differences in permissivity of porcine cell lines to APPV infection prompted us to characterize the CD46_pig_ surface expression level of these cells, as CD46_bov_ was previously shown to represent a major receptor for the related ruminant pestivirus BVDV (18). In addition, CD46_pig_ was suggested to be a major entry factor of CSFV (24). With the exception of two cell lines, immunofluorescence staining of CD46_pig_ using commercially available CD46_pig_-specific mabs revealed that most of the porcine cell lines, including SPEV and PK15, showed similar surface expression of CD46_pig_ (Fig. 2A). The only porcine cell line (38A_1_D) nonpermissive to APPV_P100_ was also the only one not expressing any CD46_pig_. A neonatal porcine tracheal cell line NPTr revealed expression of CD46_pig_ only by some clusters of cells (Fig. 2A). CD46_pig_-positive NPTr cells were particularly located in the upper layers of cells that grew in multilayer clusters. Costaining of APPV_P100_ and CD46_pig_ showed that APPV_P100_-positive cells were located only on the CD46_pig_-positive cell clusters (Fig. 2B).

Sequence analysis of CD46_pig_ obtained from SPEV and PK15 cells revealed no differences in the deduced amino acid sequences of the four ccp domains. In 38A_1_D cells, no mRNAs coding for the individual ccp domains (ccp1 to ccp4) were detectable by different RT-PCRs (Fig. 2C), demonstrating the absence of CD46_pig_ coding transcripts. This confirms the negative immunofluorescence results when staining for CD46_pig_. Taken together, these data provide strong evidence that 38A_1_D cells are deficient for CD46_pig_ expression and highlight the importance of CD46_pig_ for the APPV entry process.

### Generation and characterization of CD46_pig_ knockout cell lines.

**FIG 3:**
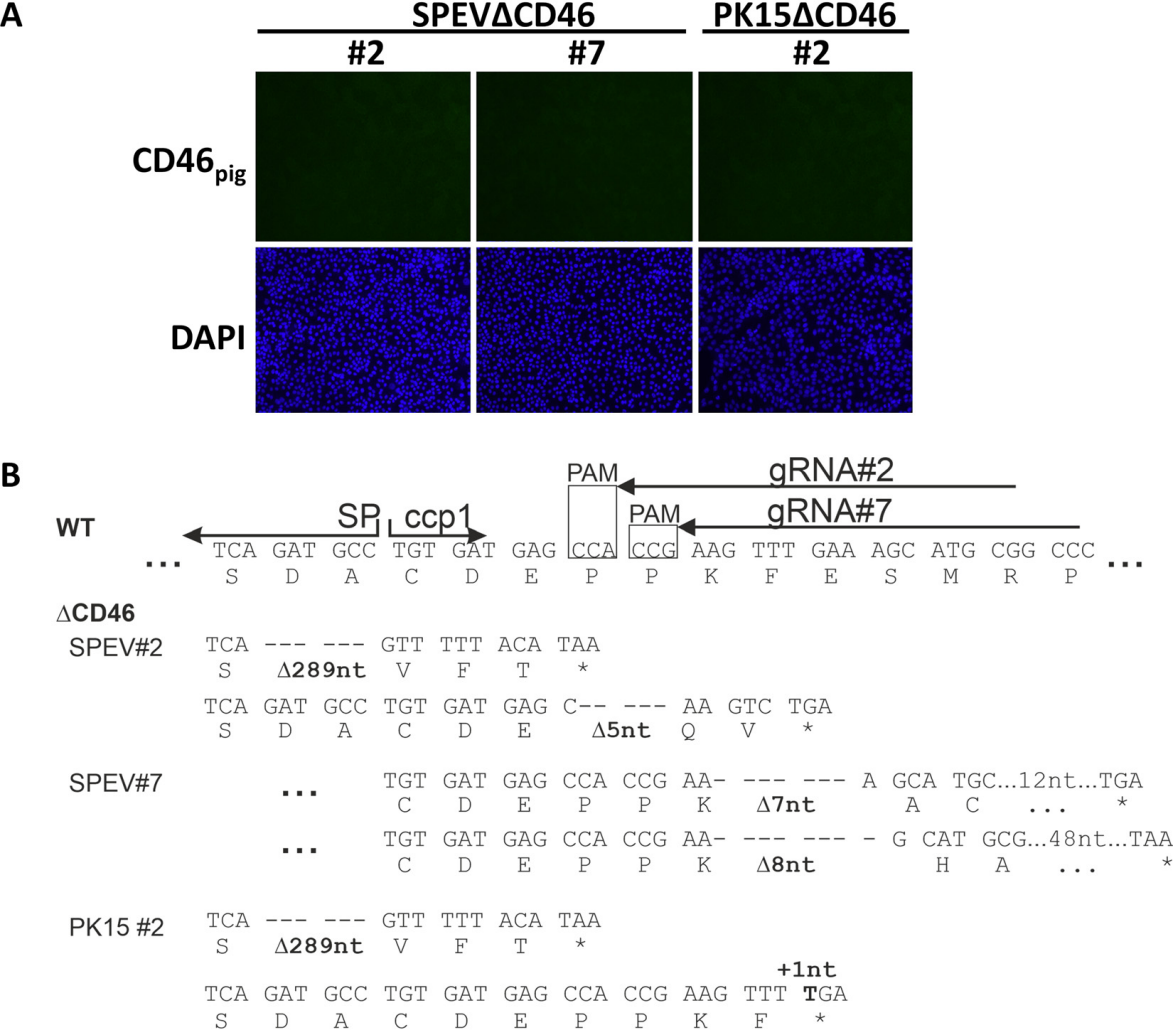
**Characterization of genetically engineered CD46_pig_ knockout cells.** (A) Phenotypical characterization of CD46_pig_ knockout cells by immunofluorescence staining using a mab against CD46_pig_ (green, MCA2310GA) and DAPI (blue). Immunofluorescence staining of CD46_pig_ (green) from wild-type (WT) cell lines served as a control and is shown in Fig. 1. (B) CRISPR/Cas9 induced genome alterations on both alleles characterized by sequencing of plasmids containing PCR amplicons flanking target sites of the guide RNAs (primers 101fw/710rev). Consensus nucleotide sequences and deduced amino acid sequences of the regions encoding the C terminus of SP and the N terminus of ccp1 are shown. For comparison, nucleotide and deduced CD46_pig_ amino acid sequences of WT as determined for SPEV and PK15 cells are given in the top row. The border between SP/ccp1 and position of gRNAs including respective protospacer adjacent motifs (PAM, boxed) are indicated. For selected engineered CD46_pig_ knockout cell lines (ΔCD46) the corresponding sequences including deletions (Δ nt) and insertions (+ nt) are shown below the WT CD46 sequence.

To investigate the CD46_pig_ dependency in the entry process of different porcine pestiviruses, CD46_pig_-deficient cell lines were generated by CRISPR/CAS9 technology and lentiviral transduction of CD46_pig_-specific guide RNAs. Two different guide RNAs (gRNAs CD46-2 and -7) were designed to avoid misinterpretation due to off-target effects (Fig. 2C, Fig. 3B, and Table 2). Both oligonucleotides were located very close to each other targeting the genomic region encoding the ccp1 domain of CD46_pig_, the receptor-binding site of CD46_bov_ for the ruminant pestivirus BVDV (26). Two different CD46_pig_ knockout SPEV cell lines (SPEVΔCD46) were obtained with gRNA CD46-2 (SPEVΔCD46 clone 2) and gRNA CD46-7 (SPEVΔCD46 clone 7), respectively (Fig. 3). Additionally, one CD46_pig_ knockout PK15 cell line was generated with gRNA CD46-2 (PK15ΔCD46 clone 2, Fig. 3) prompted by the observed differences in permissivity of wild-type (WT) SPEV and PK15 cells. All CD46_pig_ knockout cell lines were cloned biologically to obtain clonal cell lines. Immunofluorescence staining using mabs against CD46_pig_ confirmed that all generated knockout cell lines were phenotypically CD46_pig_-deficient (Fig. 3A). Sequence analysis of plasmids containing cloned PCR products of the N-terminal region of CD46_pig_ ccp1 domain confirmed different genetic alterations in this region, due to random repair mechanisms of the cell. Genetic characterization of at least 10 independent CD46_pig_ PCR plasmid clones demonstrated the presence of altered CD46_pig_ coding sequences in a diploid set of chromosomes for each cell clone. Each one of the altered alleles revealed different frameshifts within the N-terminal region of the ccp1 domain (Fig. 3B). The SPEVΔCD46 clone 2 revealed to have a small deletion of five nucleotides on one allele resulting in a stop codon and codes for only three unchanged amino acids at the N terminus of ccp1. In the other allele, an extended large deletion of 289 nucleotides was found, affecting the parts coding for the SP and the complete ccp1 sequence (195 nucleotides) as well as the N-terminal half of ccp2 (94 of 180 nucleotides, Fig. 3B). Both alleles of SPEVΔCD46 clone 7 revealed to have deletions of seven and eight nucleotides, respectively, resulting in frameshifts and stop codons (Fig. 3B). In consequence, only the first six amino acids following the signal peptide (SP) of CD46_pig_ remained unchanged in SPEVΔCD46 cell clone 7 (Fig. 3B). In the PK15ΔCD46 clone 2, in one allele, a single nucleotide insertion results in an immediate stop codon. The other allele contains the same extended deletion as observed in SPEVΔCD46 clone 2 affecting the SP, ccp1, and ccp2 sequences.

**TABLE 2:** Primers used in this study

Primer	Sequence (5′→3′)^*a*^	Target	Purpose
1539fw	CAACGTGGTCACCCAGGC	CSFV E^rns^	PCR, seq. of CSFV
2222rev	CCACCAGTCTGGTCTAACAC	CSFV E^rns^	PCR, seq. of CSFV
1114fw	GGTCTATAAGTATCCGGGAG	APPV E^rns^	PCR, seq. of APPV
1594rev	TTACCTCATCTCTAGCCTGC	APPV E^rns^	PCR, seq. of APPV
1459fw	TGGCTGGTGTAACTATCCAC	APPV E^rns^	PCR, seq. of APPV
2002rev	TACCTGAGCCAAACAGATGC	APPV E^rns^	PCR, seq. of APPV
1721fw	ATGGACGAAGCATCAATGGC	APPV E1	PCR, seq. of APPV
2251rev	TCCAAATCTGTGTAGGCCAC	APPV E1	PCR, seq. of APPV
2109fw	GATCTGAGTGGTTGGAACAC	APPV E1	PCR, seq. of APPV
2554rev	TTGGTTCCCTACCTTCCTTG	APPV E1	PCR, seq. of APPV
2353fw	CCTGGAATTAGTCTACCTGG	APPV E2	PCR, seq. of APPV
2912rev	GTAACTGGACCCATGCTTTC	APPV E2	PCR, seq. of APPV
2749fw	TTACTGGGTGAACGCAACAG	APPV E2	PCR, seq. of APPV
3324rev	AAAGCTCAAGGCTACTGGAC	APPV E2	PCR, seq. of APPV
gCD46-2fw	CACCGCGCATGCTTTCAAACTTCGG	ccp1	K.O. CD46_pig_
gCD46-2rev	AAACCCGAAGTTTGAAAGCATGCGC	ccp1	K.O. CD46_pig_
gCD46-7fw	CACCGGGCCGCATGCTTTCAAACTT	ccp1	K.O. CD46_pig_
gCD46-7rev	AAACAAGTTTGAAAGCATGCGGCCC	ccp1	K.O. CD46_pig_
pigCD46_101fw	CCCGAGAATCCCTTTTCTTC	SP	PCR, seq. of CD46_pig_
pigCD46_192fw	GTGATGAGCCACCGAAGTTTG	ccp1	PCR
pigCD46_353rev	GGGGTGACCACGTATTATCG	ccp1	PCR
pigCD46_387fw	ATCTACCAGACCCGTTAAATGGC	ccp2	PCR
pigCD46_543rev	GGCTCACTCCAGGCCATAAC	ccp2	PCR
pigCD46_571fw	TAAACCACCTGGCGAAATTCC	ccp3	PCR
pigCD46_604fw	CACCAATAGCCATAAGGATG	ccp3	PCR, seq. of CD46_pig_
pigCD46_710rev	AAAGGCTGCTCTCTCCAACA	ccp3	PCR, seq. of CD46_pig_
pigCD46_766fw	ATGTCCATATCCAGTAGTCCC	ccp4	PCR
pigCD46_937rev	GATACATTGGGGCATCTCAG	ccp4	PCR
pigCD46_1192ev	TTCCACGTCCTCTCAGCAAC	3′ NTR	PCR, seq. of CD46_pig_
T7fw	TAATACGACTCACTATAGGG	TOPO Vector	PCR, seq. of CD46_pig_
M13rev	AACAGCTATGACCATG	TOPO Vector	PCR, seq. of CD46_pig_

a Underlined sequences indicate the target site sequences (20 bp). ccp: complement control protein, SP: signal peptide, K.O.: knockout, NTR: nontranslated region.

In consequence, in addition to the 38A_1_D cell line that was identified to be naturally deficient for CD46_pig_, three genetically engineered CD46_pig_ knockout porcine cell lines were successfully generated with the aim to determine the relevance of CD46_pig_ in the entry of porcine pestiviruses.

### Relevance of CD46_pig_ for the entry of porcine pestiviruses.

A comparative analysis regarding the permissivity of WT and CD46_pig_ knockout cell lines was performed using APPV, a set of CSFV strains, and BuPV. Early (non-cell-culture-adapted APPV_P17_) and late (cell culture-adapted APPV_P100_) passages of the APPV isolate were used. Different CSFV isolates that are representatives of genotypes 1 and 2 with different virulence properties were selected, including the attenuated vaccine strain Riems (gt. 1.1), the highly virulent strain Koslov (gt. 1.1), the strains Paderborn (gt. 2.1), Diepholz (gt. 2.3), and Alfort-Tübingen (AlfT) (gt. 2.3), which was rescued from a reverse genetic system (27, 28). Sequence analysis of glycoprotein E^rns^ coding regions revealed that selected CSFV isolates do not possess a point mutation (Ser^476^ to Arg^476^) which was previously reported to be responsible for cell culture adaptation (29). CSFV isolate Paderborn naturally possesses Arg^476^ as also described earlier (GenBank ADI58615; AAL68894).

**FIG 4:**
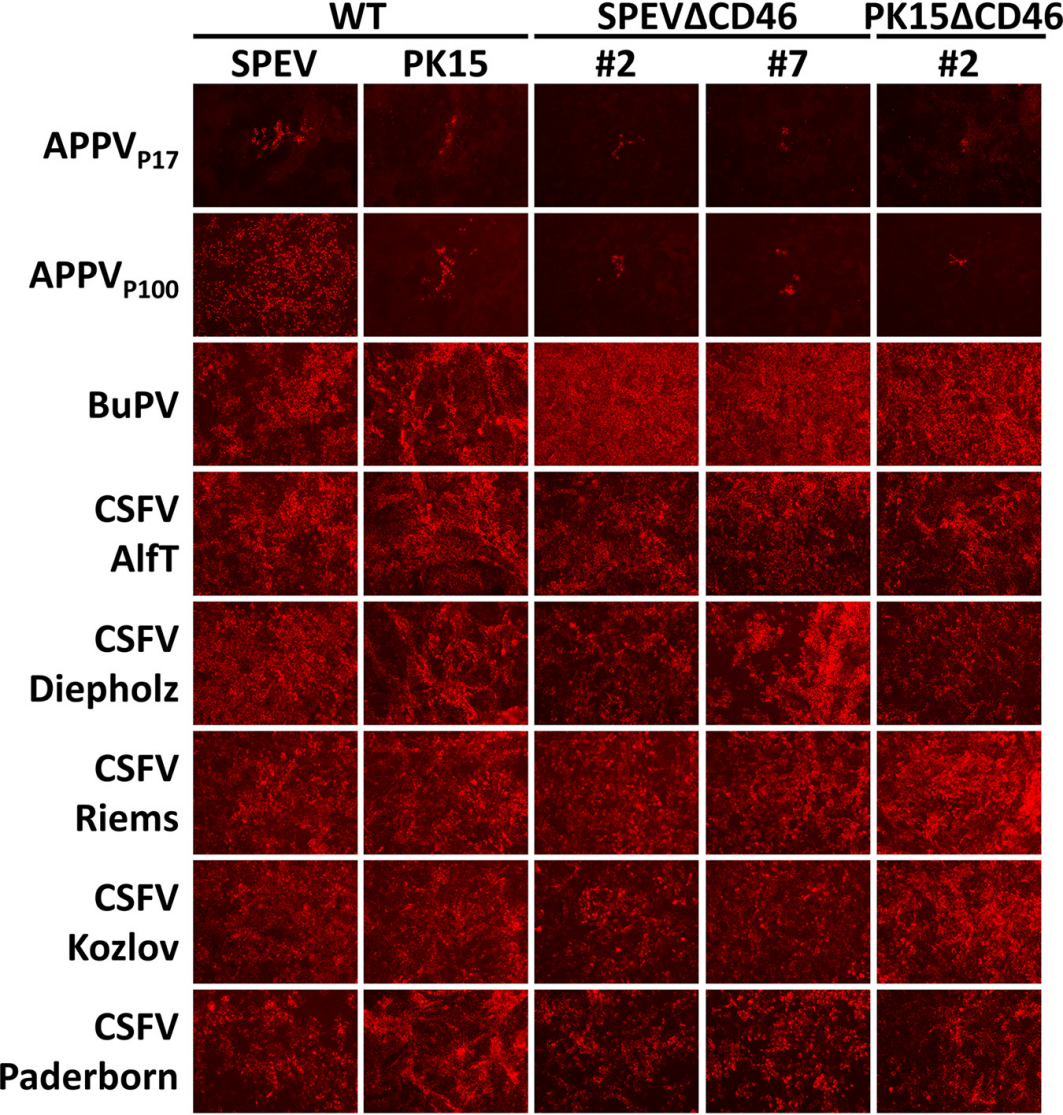
Relevance of CD46_pig_ for the entry of porcine pestiviruses. Wild-type (WT) SPEV and PK15 as well as CD46_pig_ knockout cell lines (SPEVΔCD46 clones 2 and 7 and PK15ΔCD46 clone 2) were infected with APPV_P17_, APPV_P100_, BuPV, and CSFV strains Alfort-Tübingen (AlfT), Diepholz, Riems, Koslov, and Paderborn at an MOI of 1, respectively. Immunofluorescence staining was performed at 72 h p.i. using porcine APPV-specific antiserum, a porcine BuPV-specific antiserum, and a mab against CSFV, respectively. A strong reduction of APPV infection is evident on all SPEVΔCD46 cell lines in comparison to that on SPEV cells. PK15 cells display significantly lower permissivity to APPV_P100_ compared to that of SPEV cells. Non-culture-adapted APPV_P17_ obtained from early passage revealed the same CD46_pig_ dependency as the culture-adapted variant (APPV_P100_). With regard to infections with CSFV and BuPV, there are no differences in permissivity between the WT and the CD46_pig_ knockout cell lines.

**FIG 5:**
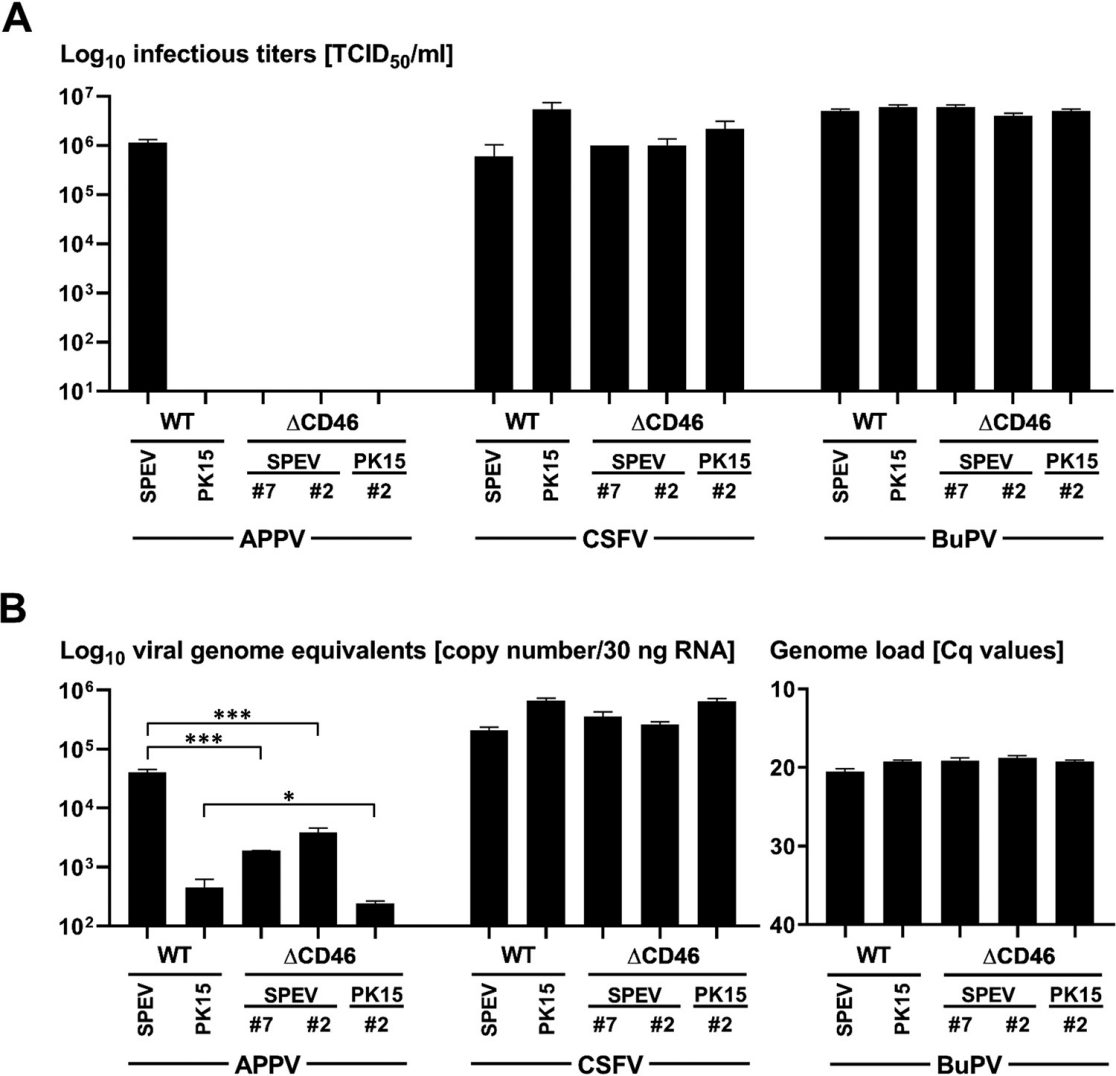
Production of infectious particles and RNA replication of porcine pestiviruses in dependence on CD46_pig_. Wild-type (WT) SPEV and PK15, as well as CD46_pig_ knockout cell lines (SPEVΔCD46 clones 2 and 7 and PK15ΔCD46 clone 2), were infected with APPV_P100_, CSFV Alfort-Tübingen (AlfT), and BuPV at an MOI of 1, respectively. (A) Supernatants were harvested 72 h p.i. to determine virus titers by using endpoint dilution assays in quadruplicates and in three repetitions. (B) Cells were collected at 72 h p.i. for RNA preparation and subsequent RT-PCR analysis. TaqMan based qRT-PCR assays were used for detection of CSFV and APPV genomes, whereas a SYBR green-based real-time RT-PCR was performed for detection of BuPV genomes. 30 ng total RNA was used per reaction. Samples collected from three individual experiments were tested in duplicates. Mean values with standard deviations are shown. APPV genome copy numbers obtained from WT cells are significantly higher compared to those from CD46_pig_ knockout cells (***, *P* < 0.0001, highly significant; *, *P* < 0.01, significant). CSFV and BuPV genome levels obtained from WT cells did not show significant differences compared to genome loads detected in infected knockout cells.

**FIG 6:**
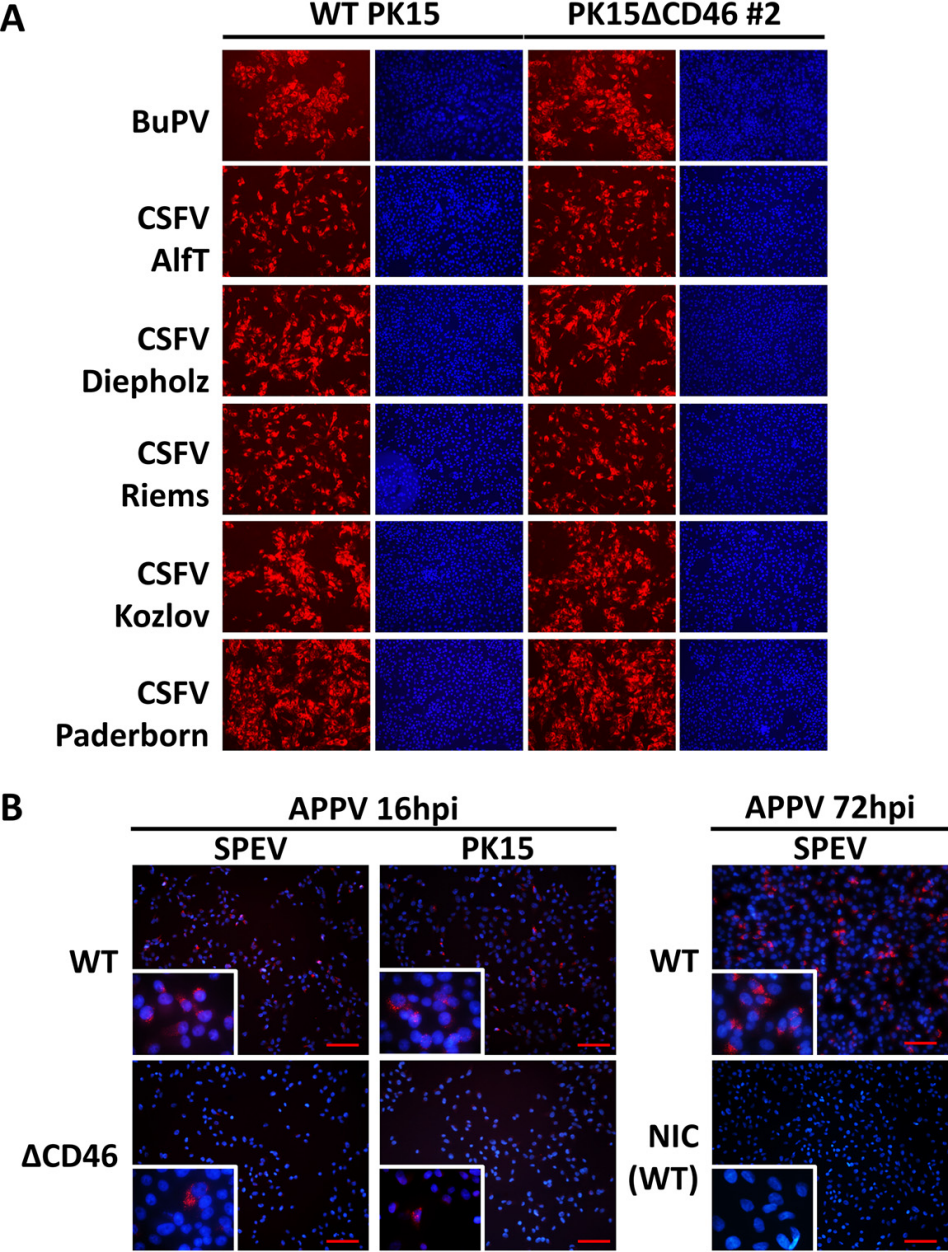
**Impact of CD46_pig_ at early time points of porcine pestivirus infections.** (A) Immunofluorescence analysis of CSFV- and BuPV-infected cells. Wild-type (WT) PK15 and PK15ΔCD46 clone 2 cells were infected with CSFV strains Alfort-Tübingen (AlfT), Diepholz, Riems, Koslov, Paderborn, and BuPV at an MOI of 1. Infections with different CSFV strains and BuPV showed no dependency on CD46_pig_ even very early after infection (16 h p.i.). (B) Fluorescence *in situ* hybridization (FISH) analysis of APPV-infected cells. WT SPEV and PK15 as well as CD46_pig_ knockout cell lines (SPEVΔCD46 clone 2 and PK15ΔCD46 clone 2) were infected with cell culture-adapted APPV_P100_ at an MOI of 0.5. Scale bars indicate 100 µm for lower magnification and 50 µm for higher magnification. A strong reduction of APPV_P100_ infection is evident on both CD46_pig_ knockout cell lines in comparison to that on WT cells at early time point of infection (16 h p.i.). APPV_P100_ genomes were observed only on single CD46_pig_ knockout cells within the infected wells. APPV_P100_ infection of CD46_pig_-expressing WT SPEV cells at a later time point (72 h p.i.) and noninfected SPEV cells (NIC) served as controls.

Infection of WT SPEV and SPEVΔCD46 cells revealed a strong impact of CD46_pig_ expression on permissivity to APPV_P100_ at 16 or 72 h p.i. (Fig. 4 and 5 and Fig. 6B). This effect was observed on both SPEVΔCD46 cell lines and confirmed the results observed with the naturally CD46_pig_-deficient 38A_1_D lymphoma cell line. Nevertheless, single small foci of APPV_P100_-infected cells were evident on both SPEVΔCD46 cell lines. Viral titers were determined 72 h p.i. (Fig. 5A). High titers were detected after *de novo* infections with APPV_P100_ reaching approximately 1 × 10^6^ 50% tissue culture infective dose (TCID_50_)/ml in the supernatant of WT SPEV cell line. In contrast, no infectious titers could be determined in the supernatants of the two different CD46_pig_ knockout cell lines. Determination of APPV_P100_ genome equivalents revealed 11 to 22 times less viral RNA in the SPEVΔCD46 cells than in WT SPEV at 72 h p.i. (Fig. 5B). In the case of PK15 cells, the dependency on CD46_pig_ for APPV_P100_ permissivity was less pronounced since the WT PK15 cell line already displayed a low permissivity comparable to that of SPEVΔCD46 cells (Fig. 4 and 5). Quantification of APPV_P100_ genome equivalents in three independent experiments revealed on average 90 times lower genome equivalents in PK15 cells than in SPEV cells (Fig. 5B). Accordingly, no infectious titers could be determined in the supernatants of the PK15 WT and knockout cell lines due to the low viral load. Interestingly, WT PK15 cells contained an approximately 10 times smaller amount of APPV_P100_ genomes than did the two SPEVΔCD46 cells, suggesting a less efficient RNA replication in PK15 cells.

Due to the lack of highly specific APPV mabs and small amounts of viral antigen early after infection, immunofluorescence staining of APPV was not possible as early as 16 h p.i. In order to perceive the impact of CD46_pig_ at an early time point of the infection, a more sensitive fluorescence *in situ* hybridization (FISH) assay was performed on APPV_P100_-infected WT and CD46_pig_ knockout cell lines (SPEVΔCD46 clone 2 and PK15ΔCD46 clone 2) at 16 h p.i. (Fig. 6B). Results of the FISH assay were in line with the immunofluorescence staining of infected cells at later time points (72 h p.i.). APPV_P100_ genomes were detected only in very few CD46_pig_ knockout cells (SPEVΔCD46 clone 2 and PK15ΔCD46 clone 2), whereas most of the CD46_pig_-positive WT SPEV and PK15 cells were highly positive for APPV_P100_ genome (Fig. 6B). Quantitative analyses of the images revealed equal amounts of positive signal in both CD46_pig_ knockout cell lines, on average (SPEVΔCD46 clone 2 and PK15ΔCD46 clone 2). Positive signals detected in WT SPEV cells were 1.9-fold higher than those in WT PK15 cells and 5.9-fold higher than those in both CD46_pig_ knockout cell lines. The difference between the WT PK15 cells and the PK15ΔCD46 2 cells was 3.1-fold.

APPV harvested from early passage 17 (APPV_P17_) showed only inefficient replication on WT SPEV and PK15 cells. Nevertheless, the impact of CD46_pig_ expression on the permissivity to APPV_P17_ was obvious when WT and CD46_pig_ knockout cells were compared (Fig. 4). Multiple infected foci were detected on WT SPEV and PK15 cells, whereas only few single infected cells were present on CD46_pig_ knockout cell lines at 72 h p.i. (Fig. 4). Due to inefficient replication of APPV, infectious titers could not be determined in the supernatants of either WT or CD46_pig_ knockout cell lines.

In contrast to APPV, no visible differences between WT and CD46_pig_ knockout cell lines were observed with regard to the permissivity to CSFV and BuPV at 16 h and 72 h p.i. (Fig. 4 and Fig. 6A). Viral titers of CSFV AlfT determined at 72 h p.i. from cell culture supernatants of infected PK15 WT and CD46_pig_ knockout cell lines were comparable (Fig. 5). Titers obtained from the infected CD46_pig_ knockout cell lines ranged between 1 × 10^6^ and 5 × 10^6^ TCID_50_/ml, while titers of 5 × 10^6^ TCID_50_/ml were determined from PK15 WT cells. Interestingly, approximately 10 times lower infectious titers of CSFV AlfT were detected in supernatants of SPEV WT cells (6 × 10^5^ TCID_50_/ml) compared to those of PK15 WT cells (Fig. 5A). Similar to CSFV titers, BuPV titers produced in WT SPEV and PK15 cell lines (5 × 10^6^ and 6 × 10^6^ TCID_50_/ml) were comparable to the viral titers produced in CD46_pig_ knockout cell lines (4 × 10^6^ to 6 × 10^6^ TCID_50_/ml). In addition, no CD46_pig_-dependent differences were observed when analyzing the amounts of viral genomes obtained from different WT and CD46_pig_ knockout cell lines infected with CSFV or BuPV at 72 h p.i., respectively (Fig. 5).

## DISCUSSION

Although pestiviruses are of outstanding importance in veterinary medicine, molecular determinants of pestiviral entry are still only poorly understood. The entry process of pestiviruses is likely to be a multistep process, as it is known to be for the related human hepatitis C virus (HCV), which uses at least four different cellular factors (30). The cellular uptake of BVDV particles is mediated by clathrin-dependent endocytosis, and it was shown to be dependent on interaction between viral E2 and cellular CD46_bov_ (14, 16, 26). For BVDV, CD46_bov_ was identified to function as the main receptor, but there are several lines of evidence indicating that an additional receptor may exist (23, 26, 31, 32).

An entry mechanism of genetically distinct pestiviruses such as APPV as well as rodent and bat pestiviruses so far could not be investigated due to lack of virus isolates and established cell culture systems. Generation of a cell culture-adapted APPV isolate (APPV_P100_) allowed us for the first time to characterize this novel pestivirus species biologically (7). In this study, reproducible genomic alterations within the predicted E^rns^ and E2 regions were identified in the culture-adapted APPV_P100_. Their appearance correlated with an increased number of infected SPEV cells (Fig. 1A). Additional alterations were also identified in the nonstructural proteins p7, NS2, NS3, NS5A, and NS5B (Fig. 1B). To clarify the relevance of observed mutations throughout the APPV genome for cell culture adaptation and to elucidate the underlying mechanisms of improved *in vitro* replication, further experiments, e.g., by reverse genetics or viral pseudotypes, will be necessary.

**FIG 7:**
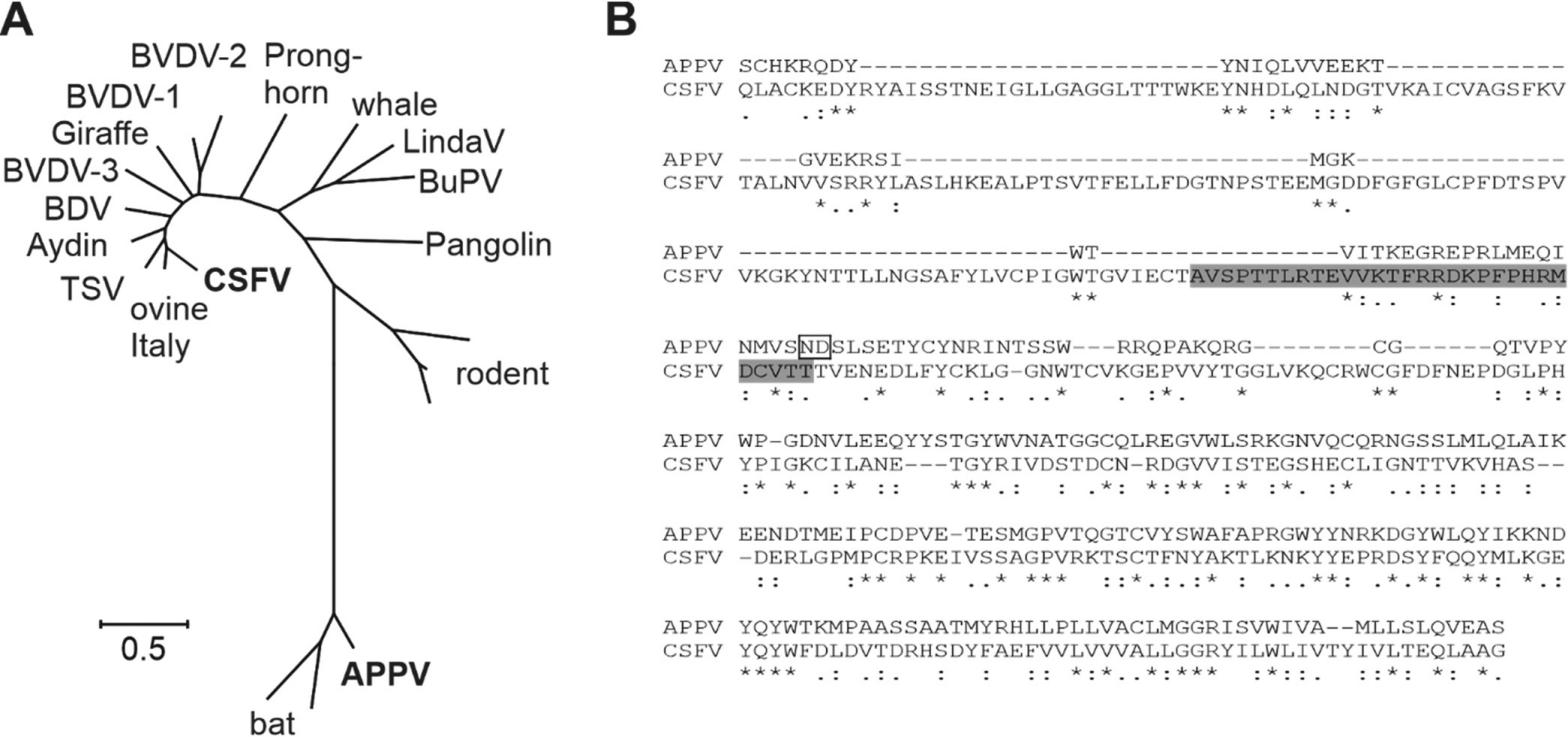
**Comparison of E2 envelope protein sequences of pestiviruses.** (A) Phylogenetic tree (maximum likelihood) based on E2 amino acid sequences of known pestivirus species (APPV: AUL76967; bat: AFK85014, AYV99177; rodent: ATP66856, ATP66857, YP009109567; pangolin: QIE06437; LindaV: YP009407716; whale: MK910228; BuPV: YP008992092; BDV: AAC16444; Aydin: YP006860588; ovine Italy: MG770617; giraffe: NP620053; pronghorn: YP009026415; BVDV-1: Q01499; BVDV-2: YP009513240; BVDV-3: AB871953; CSFV: YP009508222). APPV and CSFV sequence (bold) are the same as shown in the alignment. (B) Alignment (ClustalW) of APPV (isolate L277) and CSFV (Alfort 187) E2 amino acid sequences. Highlighted is the CSFV sequence analogous to the motif in the E2 of BVDV folding into a hairpin that might serve as ligand to the CD46_bov_ receptor (44). The positions of two nonsynonymous mutations (N751K and D752N) which occurred during cell culture adaptation of APPV are highlighted by a box.

Recently established, genetically distinct pestivirus species, including bat and rodent viruses, are apparently not restricted to ungulate hosts. Pestiviruses identified in different bat species are most closely related to APPV (Fig. 7A). The BuPV, another pestivirus species identified in pigs, shows a broad host cell tropism and is able to efficiently replicate in cells of bat and even in cells of human origin (33). Previous work showed that *in vivo* APPV differs from CSFV in tissue tropism (25). So far, host cell tropism of APPV could not be investigated due to inefficient and limited viral replication *in vitro*. The presented data revealed that APPV_P100_ obviously has a narrow host tropism and (within the cell lines used in this study) selectively infects cells of porcine origin (Fig. 1C). Different nonporcine cell lines permissive to BuPV (bat and human cells) were not permissive to APPV_P100_ (data not shown).

Investigated cell lines of porcine origin showed major differences in their permissivity to APPV_P100_ (Fig. 1C). The embryonic porcine kidney epithelial cell line SPEV allowed most efficient replication of APPV_P100_, which may explain reports of successful virus isolation only on this cell line (14, 15). Differences in permissivity to APPV_P100_ prompted us to investigate the expression of CD46_pig_. SPEV and PK15 cells, displaying major differences in permissivity to APPV_P100_, did not differ in their CD46_pig_ expression pattern. Moreover, sequence analysis did not identify differences in the regions coding for the ectodomain of CD46_pig_. Thus, the major differences in PK15 and SPEV cell permissivity to APPV_P100_ cannot be explained by genetic differences in the ccp1 domain, which was previously reported to bind glycoprotein E2 of BVDV (26). The molecular basis for restricted growth of APPV_P100_ in established porcine cell lines and differences in permissivity of CD46_pig_-expressing porcine cells should be in the focus of future studies with the aim to establish a fully permissive cell culture system. All porcine cell lines investigated in this study expressed CD46_pig_ in a uniformly distributed pattern with two exceptions (NPTr, 38A_1_D, Fig. 2A). Remarkably, neonatal porcine tracheal cell line (NPTr) revealed to express CD46_pig_ only in the upper layers of cell clusters, putatively in dependence on the differentiation status. Costaining of APPV_P100_ and CD46_pig_ in these cells indicated that CD46_pig_ expression is a prerequisite for APPV_P100_ infection (Fig. 2B). Along this line, it is an interesting finding that the porcine lymphoma cell line 38A_1_D is naturally deficient for CD46_pig_. 38A_1_D was the only porcine cell line tested in this study that was nonpermissive to APPV_P100_, and the absence of CD46_pig_ in 38A_1_D cells might be responsible for this phenotype (Fig. 1C). Oddly, previous results showed that a CSFV isolate could be grown on 38A_1_D cells to titers 10 times higher than it could on established PK15 cells (34). Thus, the finding of 38A_1_D cells to be deficient for CD46_pig_ is remarkable since CD46_pig_ was proposed to be a major receptor of CSFV entry (24).

To elucidate the role of CD46_pig_ in the entry of porcine pestiviruses, porcine knockout cell lines incapable of expressing CD46_pig_ were generated by CRISPR/Cas9, based on SPEV and PK15 cell lines. All cell lines were characterized genetically and phenotypically to be deficient for CD46_pig_ (Fig. 3). Differences in permissivity of WT and CD46_pig_ knockout cells to APPV_P17_ were observed at 72 h p.i. (Fig. 4). Moreover, with the use of culture-adapted APPV_P100_, the high impact of CD46_pig_ expression on permissivity to APPV could be observed as early as 16 h p.i. and also at 72 h p.i. (Fig. 4 and Fig. 6B). These results demonstrate that entry of APPV is highly dependent on CD46_pig_. A similar strategy based on culture adaptation was followed for other viruses that were difficult to isolate and to propagate *in vitro*, e.g., the related HCV and its culture-adapted JFH-1 clone (35). Adaptive mutations in glycoprotein regions can result in improved binding affinity of the viral ligands with attachment factors or with receptors and sometimes might even result in receptor switch. In the glycoprotein of MeV, only a single exchanged amino acid in the viral receptor ligand can be sufficient to result in a switch between usage of SLAM or nectin-4 receptors and entering the cell via human CD46 (e.g., as known for strain Edmonston). Detailed studies demonstrated that the interaction sites for the cellular receptors on the viral glycoprotein at least partially overlap and that alteration of a single amino acid can be sufficient to change receptor preference (36, 37). However, the mutations observed in the APPV glycoproteins after cell culture adaptation apparently do not result in a receptor switch since both APPV harvested from early passage (APPV_P17_) and cell culture-adapted APPV_P100_ showed comparable dependency on CD46_pig_. An increase in the affinity for CD46_pig_ in the cell culture-adapted virus is one plausible explanation. As known for heparan sulfate (HS) usage by BVDV and CSFV, improved attachment to the host cell might also help to bind to the receptor and thus result in more efficient entry. Consequently, the cell culture-adapted APPV_P100_ established in this study represents a precious tool to identify molecular determinants of entering the host cell.

In naturally infected pigs, APPV can be detected in many different tissues as reported earlier (25, 38). This is in line with the finding of this study that APPV uses the ubiquitously expressed CD46_pig_ molecule for cell entry. Nevertheless, other cellular factors are apparently implicated in the entry process and will be required to complete the viral replication cycle successfully. Therefore, it is not surprising that APPV and BVDV show some differences in tissue tropism although both are highly dependent on CD46 during entry process. It remains obscure why BVDV preferentially infects certain cell types, e.g., epithelial and immune cells. One explanation might be interaction with additional (colocalized) surface proteins (20). Very recent data demonstrated that in polarized bovine respiratory epithelial cells, CD46_bov_ is a major BVDV receptor on the apical but not the basolateral cell membranes despite the observation that basolateral infection was more efficient than apical infection (32). In consequence, the entry process of BVDV is much more complex than previously recognized. Although CD46_pig_ represents a crucial factor in APPV entry, it remains unknown which additional factors are required for efficient replication. The future identification of such host factors may also explain differences in tissue tropism of BVDV and APPV. Nevertheless, the loss-of-function experiments presented in this study clearly demonstrate an important role of CD46_pig_ during cell entry of APPV. Diverse genetic and splice variations of CD46_bov_ have been reported to shift permissivity of bovine cells to BVDV (22). Future studies need to figure out whether CD46_pig_ complementation of CD46 knockout cells in *trans* can restore the permissive phenotype of wild-type cells. Successful complementation might depend on a specific CD46_pig_ variant or availability of specific cofactors and thus can provide novel insights into molecular interaction between CD46_pig_ and APPV during the entry process.

Several cellular factors were suggested to be involved in the entry process of CSFV, including CD46_pig_, heparan sulfate (HS), laminin receptor (LamR/RPSA), the low-density lipoprotein receptor (LDLR), beta-actin, and vinculin (24, 39, 40). As antibody-mediated blocking of CD46_pig_ resulted in a reduction of CSFV infection, CD46_pig_ was proposed to be a main receptor of CSFV, along with the attachment factor HS and possibly other unknown receptors (24). However, the inhibitory effect of anti-CD46_pig_ antibodies may rely on steric side effects interfering with virus/receptor interaction. In a different study, blocking of the CD46_bov_ receptor with an antiserum provided no evidence for involvement in entry of CSFV, BDV, or Giraffe pestivirus (26). Importantly, amino acid composition of the BVDV E2 binding motif (E_66_QIV_69_ and G_82_QVLAL_87_) of CD46_bov_ and the analogous region in CD46_pig_ of porcine cells display major differences (26). The data obtained from infecting the well-defined porcine CD46_pig_ knockout cells clearly show that, in contrast to APPV, CD46_pig_ is not essential for cell entry of the porcine pestiviruses CSFV and BuPV (Fig. 4 and 5 and Fig. 6A). Surprisingly, CSFV and BuPV seem to use an alternative route of cell entry. Cell-to-cell spread may result in overcoming the CD46_pig_ receptor usage via engagement of so far unknown alternate receptors on the target cell (41). Nevertheless, even as early as 16 h p.i., avoiding prominent effects of cell-to-cell transmission, no impact of CD46_pig_ on CSFV or BuPV infection became evident (Fig. 6A). A specific mutation in the E^rns^ protein of CSFV (Ser^476^ to Arg^476^) was previously reported to result in cell culture adaptation via improved attachment to the cell by increasing the affinity to HS (29, 42). CSFV isolates used in this study, including the recombinant CSFV strain AlfT as well as isolates Riems, Koslov, and Diepholz, do not possess this specific mutation and thus are not adapted to efficient HS attachment. All isolates were able to infect the CD46_pig_-deficient knockout cells very efficiently. This strongly suggests that HS usage is not implicated in efficient entry of these CSFV strains (Fig. 4 and Fig. 6A). In consequence, it is likely that a so far unknown receptor is used by CSFV to enter the cells. The representative selection of CSFV isolates (different genotypes and virulence properties) used in this study provide evidence that CD46_pig_-independent entry of CSFV is not restricted to a particular CSFV isolate.

Glycoprotein E2 of classical pestivirus species is the only known viral determinant possessing receptor-binding properties. However, simple transfer of BuPV E2 protein was not sufficient to expand host cell range of BVDV to human cells which are susceptible to BuPV (33). Interestingly, amino acid composition and predicted size of E2 glycoproteins of classical and recently discovered atypical pestiviruses display major differences (Fig. 7A). These differences can be expected to result in altered protein structure and different protein-protein interactions and may correlate with different mechanisms of cellular entry (43). There is strong evidence that a stretch of amino acids in domain II of pestivirus E2 functions as the receptor ligand (amino acids 834 to 863 in BVDV strain NADL, PDB 4JNT). This motif is exposed as a hairpin in the crystal structure of BVDV E2 (44). The distinct E2 amino acid composition of APPV did not allow for identification of a corresponding receptor-binding motif (Fig. 7B). Against this background, it is not surprising that the distantly related porcine pestiviruses APPV and CSFV use different strategies of cell entry. Remarkably, BVDV and APPV obviously share the same receptor, although they are only distantly related and infect different host species (ruminant and porcine, respectively).

Taken together, the presented results demonstrate that CD46_pig_ is a major cellular factor for efficient entry of APPV. With regard to its role in viral entry, the function of CD46_pig_ for APPV infection resembles the function of CD46_bov_ for BVDV entry. Additionally, our study demonstrates that (in contrast to BVDV and APPV) other porcine pestiviruses like CSFV and BuPV apparently use a CD46_pig_-independent way to enter the host cell. This indicates that CD46 is not a general receptor of pestiviruses and that different pestivirus species apparently use diverse mechanisms for host cell entry.

## MATERIALS AND METHODS

### Cells and viruses.

The porcine kidney cell lines SPEV and PK15 were maintained as a monolayer in Earle’s minimal essential medium (EMEM) containing 5% fetal bovine serum (FBS) for the infection experiments and generation of the knockout cell lines. Other cell lines were maintained as a monolayer in Dulbecco’s modified Eagle medium (DMEM) containing either 10% FBS or horse serum (MDBK and CRFK) (Table 1).

Selected CSFV strains include representatives of the most relevant genotypes 1 and 2. CSFV strains Alfort-Tübingen (AlfT, CSF0904, genotype 2.3), Diepholz (CSF0104, genotype 2.3), Paderborn (CSF0277, genotype 2.1), Koslov (CSF0382, genotype 1.1), and Riems (CSF0913, vaccine strain, genotype 1.1) were obtained from the virus collection (CSF catalogue numbers are given in parentheses) of the Institute of Virology, University of Veterinary Medicine Hannover, Germany (45). These CSFV strains show differences in virulence properties, including the attenuated vaccine strain Riems, a variant of the live attenuated C-strain, and the extraordinary virulent strain Koslov, which is often used in vaccination-challenge studies. Glycoprotein E^rns^ coding regions were monitored for a point mutation (Ser^476^ to Arg^476^) which was previously reported to be responsible for cell culture adaptation via heparan sulfate (HS) usage (29). RT-PCR and subsequent Sanger sequencing were performed using primer pair 1539fw/2222rev (Table 2).

BuPV was kindly provided by Peter Kirkland (Elizabeth Macarthur Agriculture Institute, Menangle, Australia). Virus stock from the 17th passage of the APPV isolate Ger-NRW_L277 (GenBank MF167291) represents the non-cell-culture-adapted APPV stock (APPV_P17_). Virus stock from the 100th passage of APPV (APPV_P100_) was obtained as previously described, showing a viral titer of approximately 8 × 10^4^ TCID_50_/ml on SPEV cells (7).

Increasing numbers of APPV-positive cells were observed during continuous passaging of persistently infected SPEV cells. For characterization of the cell culture adaptation, naive SPEV cells were infected with 1 ml APPV cell culture supernatant from the 25th, 45th, 55th, and 100th passage in six-well plates at the time point of seeding. Cells were heat fixed 72 h p.i. for immunofluorescence staining as described below. Viral titers were determined from the supernatants of 25th, 55th, and 100th passages of APPV. Genomic alterations within the predicted glycoprotein-encoding regions were monitored after the 17th, 25th, 35th, 45th, 55th, and 100th passages of APPV by RT-PCR and subsequent Sanger sequencing, using primer pairs 1114fw/1594rev, 1459fw/2002rev, 1721fw/2251rev, 2109fw/2554rev, 2353fw/2912rev, and 2749fw/3324rev (Table 2). Moreover, at the 100th passage (APPV_P100_), the complete polyprotein coding sequence was determined using next-generation sequencing as previously described (25).

### Permissivity of different cell lines to atypical porcine pestivirus (APPV).

One day prior to infection, porcine cell lines SPEV, PK15, SK6, NPTr, IPECJ2, ST, and 38A_1_D and nonporcine cell lines MDBK, CRFK, RK13, Vero76, Lovo, HypNi/1.1, and EidNi/41 were seeded in 24-well plates. On the day of infection, cells from two wells of each plate were trypsinized and counted. All cells were infected with a multiplicity of infection (MOI) of 1 with APPV_P100_. Cells were incubated at 37°C for 72 h, heat fixed at 80°C (4 h), and analyzed by APPV-specific immunofluorescence. Immunofluorescence staining was performed using porcine APPV-specific antiserum (dilution 1:2,000) in combination with secondary mab Alexa fluor 594 goat anti-swine IgG (111-585-003, Dianova, 1:1,000 dilution).

### Generation of CD46_pig_ knockout cell lines.

Generation of knockout cells by CRISPR/Cas9 technology was performed as described previously (46). Briefly, oligonucleotides containing the guide sequences (primers gCD46-2 and gCD46-7) targeting the region encoding the N-terminal part of complement control protein 1 (ccp1) within CD46_pig_ were designed (Table 2) and cloned into the plentiCRISPR-v2 plasmid to generate plentiCRISPR-v2-CD46-2 and plentiCRISPR-v2-CD46-7 (47, 48). For production of lentiviral particles, the recombinant plasmids were cotransfected with a packaging vector (pCMVΔR8.91) and a plasmid encoding the glycoprotein of vesicular stomatitis virus (VSV-G-pMD.G) into HEK293T cells, using polyethylenimine transfection reagent (24765-1, Polysciences, Inc.). Harvested lentiviral particles were used for transduction of SPEV and PK15 cells, respectively, followed by a subsequent puromycin selection (P8833, Sigma-Aldrich) with 5 µg/ml puromycin-supplemented medium for 2 weeks. At least three rounds of biological cloning of SPEVΔCD46 and PK15ΔCD46 cell lines were performed by single cell expansion. Purity of the obtained knockout cell lines was confirmed by immunofluorescence analysis and genetic characterization as described below.

### Phenotypic and genetic characterization of wild-type porcine cell lines and CD46_pig_ knockout cells.

Presence of CD46_pig_ in wild-type (WT) porcine cell lines as well as in individual knockout cell clones was evaluated by immunofluorescence staining. Cells were grown for 3 days and heat fixed at 80°C (4 h). Staining was performed using commercially available mabs against CD46_pig_ (MCA2310GA and MCA2262GA, Bio-Rad, 1:500 dilution) and a secondary mab Alexa fluor 488 goat anti-mouse IgG (A11029, Invitrogen, 1:1,000 dilution). Additionally, cell nuclei were stained with DAPI ([4′,6-diamidino-2-phenylindole]; D3571, ThermoFisher Scientific, 1:500 dilution).

For genetic characterization of CD46_pig_ expressed by parental SPEV and PK15 cells, RNA preparations were used for reverse transcription by SuperScript III reverse transcriptase (18080093, Invitrogen) and random hexamers (N8080127, Invitrogen). Amplification of a partial CD46_pig_ coding sequence was performed using ALLin HS Red Tag Mastermix (HSM0305, highQu) with two primer pairs (101fw/710rev and 604fw/1192rev, Table 2) for subsequent generation of a consensus sequence (1,092 bp). The following thermal profile was applied: 2 min at 95°C, 40 cycles; 15 s at 95°C, 15 s at 54°C, 15 s at 72°C, and final extension for 5 min at 72°C. Sanger sequencing was conducted by LGC genomics (Berlin, Germany). Attempts to amplify the CD46_pig_ coding sequence from 38A_1_D cells with these primers were not successful. Thus, four additional primer pairs were designed, which target genetic regions representing the individual domains ccp1 to ccp4 of CD46_pig_ (192fw/353rev, 387fw/543rev, 571fw/710rev, 766fw/937rev; Table 2).

To characterize the CRISPR/Cas9-induced genome alterations in the engineered CD46_pig_ knockout cells, primers (101fw/710rev) flanking the target site of the guide RNAs were applied. These primers were specific for regions coding for the signal peptide and the ccp3 domain, respectively (Table 2). Gel purified amplicons (wild-type genome: 610 bp) were cloned into TOPO vector (450030, ThermoFisher Scientific) and propagated in TOP10 E. coli bacteria. Individual colonies were subjected to conventional PCR using vector specific primers T7fw and M13rev (Table 2). Subsequently, amplicons of at least 10 individual colonies were subjected to Sanger sequencing (LGC genomics, Berlin, Germany) to determine genetic variation within the individual cell clones. Obtained sequences were analyzed using the GENtle software (version 1.9.4.0) and compared to the CD46_pig_ coding sequences of SPEV and PK15 WT cells.

### Infection of wild-type and CD46_pig_ knockout cell lines.

Titers of virus stocks of APPV_P100_, BuPV, and different CSFV strains were determined, and virus stocks were stored at –80°C until use for infection experiments. Titer of virus stock APPV_P17_ could not be determined due to inefficient viral replication. Titers were determined by three independent endpoint dilution assays each performed in quadruplicates. Visualization of viral infection was done by immunofluorescence staining.

One day prior to infection, 3 × 10^4^ SPEV, PK15, and CD46_pig_ knockout cells (SPEVΔCD46 clones 2 and 7 and PK15ΔCD46 clone 2) were seeded into 24-well plates. Cells were infected with 500 µl of APPV_P17_ since the viral titer is unknown. Infections with APPV_P100_, BuPV, and the different CSFV strains were performed at an MOI of 1 for 2 h at 37°C, respectively. Subsequently, inoculum was removed and infected cells were washed three times with PBS and incubated for 72 h at 37°C. Supernatant was harvested to determine the virus titer at 72 h postinfection. Cells were washed three times with PBS and subsequently analyzed by immunofluorescence staining or RT-PCR analysis. Additionally, infections of WT and CD46_pig_ knockout cell lines with CSFV and BuPV (MOI of 1, described above) as well as with APPV_P100_ (MOI of 0.5) were analyzed 16 h postinfection (p.i.) by either immunofluorescence staining (CSFV and BuPV) or fluorescence *in situ* hybridization (FISH) assay (APPV). For the FISH assay, 1 day prior to the APPV infection, 4 × 10^5^ SPEV, PK15, and knockout SPEVΔCD46 clone 2 and PK15ΔCD46 clone 2 cells were seeded on Nunc Lab-Tek II CC2 chamber slides (S6690, Merck). All infection experiments were repeated three times.

### Detection of viral replication by immunofluorescence or fluorescence *in situ* hybridization assay.

For immunofluorescence staining, cells were heat fixed at 80°C (4 h). Immunofluorescence staining was performed using a porcine APPV-specific antiserum (dilution 1:2,000) or a porcine BuPV-specific antiserum (dilution 1:12,000) in combination with secondary antibody Alexa fluor 594 goat anti-swine IgG (111-585-003, Dianova, 1:1,000 dilution), respectively. For visualization of CSFV infection, pestivirus-specific mab C16 (dilution 1:50) was used together with secondary antibody Cy3-AffiniPure goat anti-mouse IgG (115-165-146, Dianova, 1:800 dilution) (49).

As immunofluorescence staining was not suitable to detect APPV infection at an early time point of infection (16 h p.i.), FISH assay was performed. The assay was optimized for the cell lines used in this study based on the manufacturer’s protocol (QVC0001, ThermoFisher Scientific). Formaldehyde fixation was applied for 30 min. Cy3 labeled APPV-specific probes were based on the sequence of APPV isolate L277 (GenBank MF167291) and located within the NS3 to NS4B region (3958-7236 base sequence, ViewRNA Type1 probe set, ThermoFisher Scientific). Fluorescein isothiocyanate (FITC)-labeled pig β-actin-specific probes were used as an internal reference (ViewRNA Type4 probe set, ThermoFisher Scientific).

Quantitative analyses of the images, which were generated from three independent FISH assays, were carried out using ImageJ software (version 1.51.0). The APPV-specific staining image was converted to a mask by implementing the Renyi Entropy thresholding method. APPV-specific signal is determined using the pixel count tool in histogram.

### Determination of viral replication by RT-PCR assay.

For RT-PCR analysis, cells were lysed and collected using an RA1 buffer (740961.500, Macherey-Nagel). RNA extraction was performed with KingFisher Duo Prime instrument (ThermoFisher Scientific) using IndiMag Pathogen kit (SP947257, Indical Bioscience). RNA amounts were measured using NanoDrop 2000 (ThermoFisher Scientific) and adjusted to 30 ng per reaction. TaqMan based qRT-PCR assays were used for detection of CSFV and APPV genomes (25, 50). For detection of BuPV genomes, previously established SYBR green-based real-time PCR (RT-PCR) using a primer pair (LinBu) targeting part of the NS5B coding region was performed (9). All samples were tested in duplicates using the Mx3005P QPCR system (Agilent Technologies, Santa Clara, USA) and the QuantiTect SYBR green RT-PCR kit or QuantiTect Probe RT-PCR kit (204245, 204445, Qiagen) according to the manufacturer’s protocols. The housekeeping gene coding for GAPDH was used in all RT-PCR assays for normalization. Statistical analyses were carried out using an unpaired *t* test implemented in GraphPad Prism software (version 8.4.3).
